# Effects of High-Intensity Interval Training vs. Sprint Interval Training on Anthropometric Measures and Cardiorespiratory Fitness in Healthy Young Women

**DOI:** 10.3389/fphys.2018.01738

**Published:** 2018-12-05

**Authors:** João Pedro A. Naves, Ricardo B. Viana, Ana Cristina S. Rebelo, Claudio Andre B. de Lira, Gustavo D. Pimentel, Patrícia Cristina B. Lobo, Jordana C. de Oliveira, Rodrigo Ramirez-Campillo, Paulo Gentil

**Affiliations:** ^1^Department of Physical Education, Faculty of Physical Education and Dance, Federal University of Goiás, Goiânia, Brazil; ^2^Department of Morphology, Biological Sciences Institute, Federal University of Goiás, Goiânia, Brazil; ^3^Clinical and Sports Nutrition Research Laboratory, Nutrition Faculty, Federal University of Goiás, Goiânia, Brazil; ^4^Laboratory of Measurement and Assessment in Sport, Department of Physical Activity Sciences, Research Nucleus in Health, Physical Activity and Sport, Universidad de Los Lagos, Osorno, Chile

**Keywords:** interval training, exercise, physical fitness, weight loss, cardiorespiratory fitness

## Abstract

**Purpose:** To compare the effects of 8 weeks of two types of interval training, Sprint Interval Training (SIT) and High-Intensity Interval Training (HIIT), on anthropometric measures and cardiorespiratory fitness in healthy young women.

**Methods:** A randomized clinical trial in which 49 young active women [age, 30.4 ± 6.1 years; body mass index, 24.8 ± 3.1 kg.m^−2^; peak oxygen consumption (VO_2_peak), 34.9±7.5 mL.kg^−1^.min^−1^] were randomly allocated into a SIT or HIIT group. The SIT group performed four bouts of 30 s *all-out* cycling efforts interspersed with 4 min of recovery (passive or light cycling with no load). The HIIT group performed four bouts of 4-min efforts at 90–95% of peak heart rate (HRpeak) interspersed with 3 min of active recovery at 50–60% of HRpeak. At baseline and after 8 weeks of intervention, waist circumference, skinfolds (triceps, subscapular, suprailiac, abdominal, and thigh), body mass and BMI were measured by standard procedures and cardiorespiratory fitness was assessed by cardiorespiratory graded exertion test on an electromagnetically braked cycle ergometer.

**Results:** The HIIT and SIT groups improved, respectively, 14.5 ± 22.9% (*P* < 0.001) and 16.9 ± 23.4% (*P* < 0.001) in VO_2_peak after intervention, with no significant difference between groups. Sum of skinfolds reduced 15.8 ± 7.9 and 22.2 ± 6.4 from baseline (*P* < 0.001) for HIIT and SIT groups, respectively, with greater reduction for SIT compared to HIIT (*P* < 0.05). There were statistically significant decreases in waist circumference (*P* < 0.001) for the HIIT (−3.1 ± 1.1%) and SIT (−3.3 ± 1.8%) groups, with no significant difference between groups. Only SIT showed significant reductions in body weight and BMI (*p* < 0.05).

**Conclusions:** Eight weeks of HIIT and SIT resulted in improvements in anthropometric measures and cardiorespiratory fitness, even in the absence of changes in dietary intake. In addition, the SIT protocol induced greater reductions than the HIIT protocol in the sum of skinfolds. Both protocols appear to be time-efficient interventions, since the HIIT and SIT protocols took 33 and 23 min (16 and 2 min of effective training) per session, respectively.

## Introduction

Interval training (IT) has been used for many decades with different purposes, such as improvements to health parameters (Wisløff et al., 2009; Kemi and Wisløff, 2010; Weston et al., 2013), performance (McMillan et al., 2005; Gibala and McGee, 2008; Gibala and Jones, 2013), and weight loss (Trapp et al., 2008; Boutcher, 2011). Typically, IT implicates alternating periods of relatively intense exercise with periods of lower-intensity effort or complete rest for recovery (Gibala et al., 2014). Two of the most common forms of IT are high-intensity interval training (HIIT) and sprint interval training (SIT) (Gibala et al., 2014). The target intensity during HIIT is usually “near maximal” or between 80 and 100% of maximal heart rate (HRmax) or maximum oxygen consumption (VO_2_max), while SIT protocols usually involve “*all-out*” efforts (Buchheit and Laursen, 2013).

Regarding the applications for weight loss, a review found that fat loss after IT was greater than that after moderate-interval continuous training (MICT) (60–80% of HRmax) (Boutcher, 2011). Moreover, studies on the effects of IT on post exercise energy expenditure and fat oxidation (Treuth et al., 1996; Laforgia et al., 1997; Greer et al., 2015) and weight loss (Tremblay and Bouchard, 1994; Trapp et al., 2008; Burgos et al., 2017) suggest that IT is more efficient than continuous models, including MICT (Zhang et al., 2017). In fact, weight loss seems to be higher, even if the caloric expenditure obtained with IT is lower than (Tremblay and Bouchard, 1994) or equal to that of MICT (Trapp et al., 2008). These results can be attributed to the effects of IT on metabolism, promoting increased resting energy expenditure and fat utilization (Kiens and Richter, 1998; Knab et al., 2011; Kelly et al., 2013). Moreover, it seems that fat loss is greater at higher exercise intensities (Tremblay and Bouchard, 1994). However, we were not able to find any study in relation to the effects of SIT vs. HIIT on body composition in healthy young women.

Considering the meaningful differences between IT variations (Viana et al., 2018), it is important to analyze each protocol in detail to get further insight on how variations would be more suitable for a specific purpose in a given population. Two of the most popular types of interval training protocol are those presented by the Wisløff group (Wisløff et al., 2007) and the Gibala group (Gibala et al., 2006), which can be classified as HIIT and SIT, respectively. Such protocols have gained notoriety for inducing cardiovascular and performance adaptations equal to or greater than those induced by MICT despite the lower volume of exercise. However, despite their popularity, the effects of these protocols on markers of body fatness need more clarification, and we are not aware of any comparison between them. Thus, the aim of the present study was to compare the effects of two types of IT (HIIT and SIT) on anthropometric measures and cardiorespiratory fitness in healthy young women.

## Materials and Methods

### Study Design

The participants performed a HIIT or SIT protocol on a mechanically braked cycle ergometer (Evolution SR, Schwinn, USA) three times per week (Monday, Wednesday, and Friday) for 8 weeks. One week before and 1 week after the intervention, anthropometric evaluation, and a cardiorespiratory graded exertion test (GXT) on a cycle ergometer were performed. The volunteers were asked to not perform any other exercise activity apart from the study protocol. The HIIT and SIT sessions lasted 33 and 23 min, respectively. Due to the nature of the interventions, it was not possible to blind the participants and supervisors involved in the study. However, all assessments were completed by blinded technicians. When a participant missed fewer than three training sessions non-consecutively, the sessions were replaced at the end of the period, but when three or more sessions were missed, the participant was excluded from the study.

Participants were advised to maintain their usual diet. Six 24-h dietary recalls were performed at the beginning and end of the intervention.

### Participants

**Figure 1:**
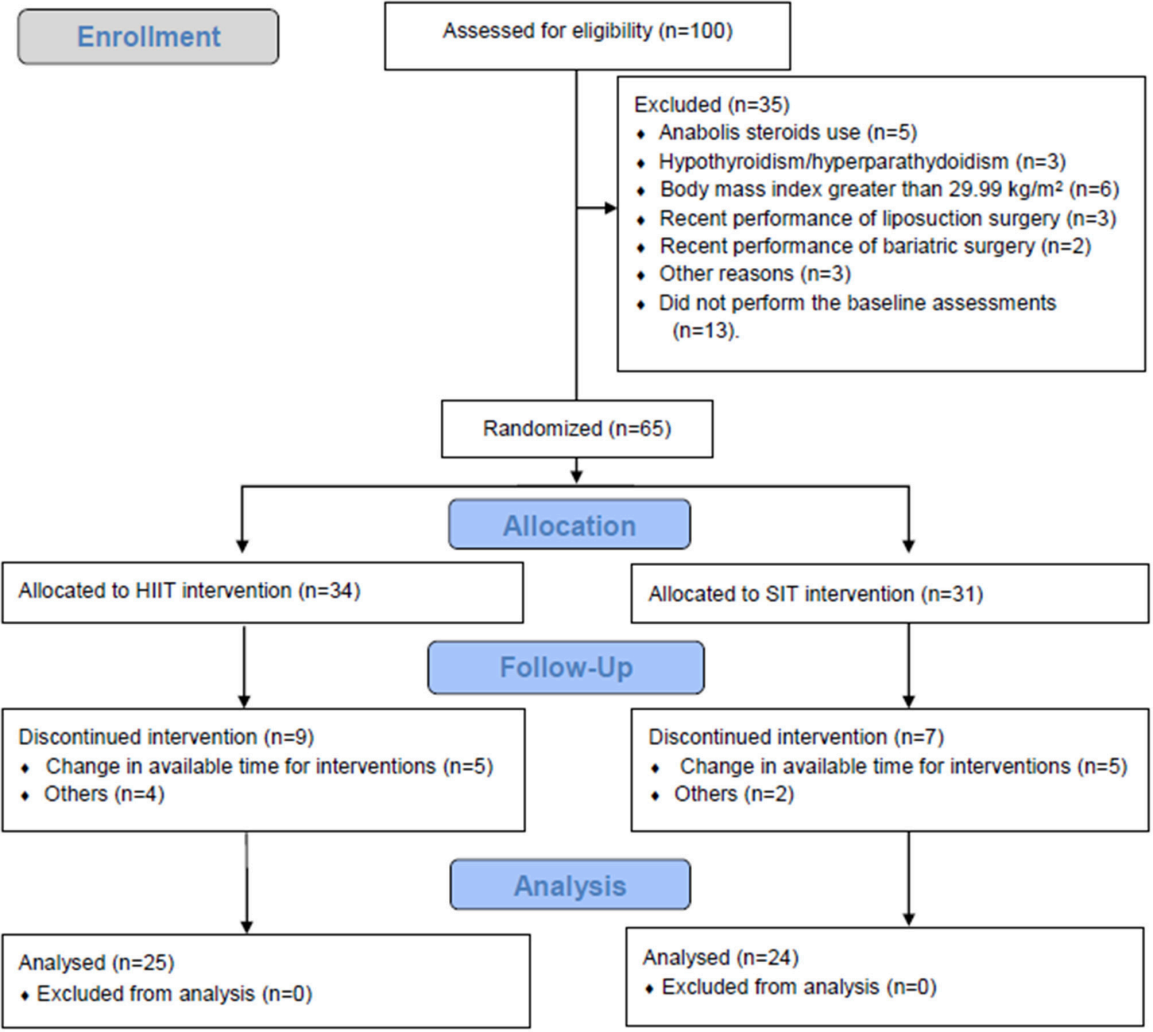
Flow diagram of participants through all phases of the trial.

Forty-nine healthy women (Table 1) were recruited through advertisements on social media and through word of mouth. The following inclusion criteria were adopted: (i) body mass index (BMI) between 18.5 and 29.9 kg.m^−2^, (ii) physically active (≥150 min per week), (iii) pre-menopause, and (iv) not using stimulants (e.g., caffeine, energy drinks, or thermogenic drugs). Exclusion criteria were (i) contraindications to physical activity assessed through the *Physical Activity Readiness Questionnaire*—PAR-Q (Canadian Society for Exercise Physiology, 2002) and (ii) any history of interventions for body mass loss (surgical or hormonal treatment). Figure 1 shows the flow diagram with all reasons for participants' exclusion and abandonment of the intervention.

**Table 1:** Anthropometric and physiological characteristics of participants before and after 8 weeks of exercise training.

	**HIIT (*****n*** = **25)**				**SIT (*****n*** = **24)**				**Between groups (Pre)**	**Between groups (Pre-Post)**
	**Pre**	**Post**	**ES**	***P***	**Pre**	**Post**	**ES**	***P***	***P***	***P***
Age (years)	31.0 ± 6.0	–	–	–	29.8 ± 6.4	–	–	–	0.823	–
Height (m)	1.63 ± 0.05	–	–	–	1.64 ± 0.05	–	–	–	0.814	–
HRpeak (beats/min)	182 ± 10	–	–	–	179 ± 13	–	–	–	0.021	–
Body mass (kg)	66.3 ± 10.2	65.9 ± 9.9	−0.039_(trivial)_	0.280	67.8 ± 8.1	67.0 ± 8.1	−0.098_(trivial)_	0.015	0.156	0.360
Body mass index (kg.m^−2^)	24.5 ± 3.3	24.4 ± 3.2	−0.030_(trivial)_	0.402	25.2 ± 3.2	24.9 ± 3.3	−0.092_(trivial)_	0.019	0.950	0.293
Skinfolds (mm)									
Triceps	22.0 ± 6.3	18.8 ± 5.2	−0.553_(medium)_	< 0.001	27.6 ± 7.7	22.8 ± 5.6	−0.712_(medium)_	< 0.001	0.213	0.909
Subscapular	21.2 ± 9.9	17.3 ± 7.3	−0.448_(small)_	< 0.001	27.4 ± 8.6	20.3 ± 5.8	−0.967_(large)_	< 0.001	0.393	0.074
Suprailiac	21.0 ± 11.2	17.4 ± 8.8	−0.357_(small)_	< 0.001	30.4 ± 8.3	23.2 ± 5.3	−1.033_(large)_	< 0.001	0.084	0.374
Abdominal	25.0 ± 11.2	19.7 ± 8.5	−0.533_(medium)_	< 0.001	35.5 ± 6.9	25.8 ± 4.7	−1.643_(large)_	< 0.001	0.029	0.111
Thigh	32.7 ± 8.6	28.2 ± 7.7	−0.551_(medium)_	< 0.001	38.3 ± 7.9	30.7 ± 6.8	−1.031_(large)_	< 0.001	0.883	0.020
∑skinfolds (mm)	121.9 ± 43.8	101.4 ± 34.6	−0.519_(medium)_	< 0.001	159.1 ± 35.1	122.8 ± 24.8	−1.194_(large)_	< 0.001	0.310	0.045
Waist circumference (cm)	74.6 ± 8.0	72.3 ± 7.8	−0.291_(small)_	< 0.001	77.6 ± 7.0	75.1 ± 6.8	−0.362_(small)_	< 0.001	0.483	0.739
VO_2_peak (mL.kg^−1^.min^−1^)	37.7 ± 7.2	42.1 ± 5.5	0.686_(medium)_	< 0.001	32.0 ± 7.2	36.5 ± 6.7	0.647_(medium)_	< 0.001	0.860	0.097
*i*VO_2_peak (watts)	159 ± 31	167 ± 27	0.275_(small)_	0.028	138 ± 26	149 ± 20	0.474_(small)_	0.003	0.303	0.402

*Data are expressed as means ± standard deviation. p (values) for within-group (time) effect and interaction (time × group) effect. ES, effect size; HIIT, high-intensity interval training; SIT, sprint interval training; HRpeak, peak heart rate; ∑Skinfolds, sum of five skinfolds; VO_2_peak, peak oxygen uptake; iVO_2_peak, intensity associated to peak oxygen uptake*.

All participants were informed of the potential risks and benefits of the study and signed an informed consent form. All experimental procedures were approved by the University Ethics Committee (Approval number: 1.542.353). The study conformed to the principles outlined in the Declaration of Helsinki.

### Interval Training Intervention

During the study period, the participants were requested to avoid any form of physical activity besides the study protocols. The research team constantly monitored and questioned the participants to verify if they complied with the recommendations and to record any adverse event (dizziness, nausea, muscle soreness…). The intervention lasted 8 weeks, with three sessions per week (Monday, Wednesday, and Friday).

The SIT group performed a warm-up of 5 min at light load and self-selected speed, followed by four 30-s all-out bouts interspersed with 4 min of recovery (passive or light cycling with no load). If necessary, the load was adjusted to allow the participant to maintain cycling cadence ≥ 60 rpm.

The HIIT protocol consisted of a warm-up of 5 min at 50% of peak heart rate (HRpeak) (FT1, Polar, Finland), followed by four bouts of 4-min efforts at 90–95% of HRpeak interspersed with 3 min of active recovery at 50–60% of HRpeak. The load was adjusted when the HR deviated from the established zone. During recovery, the cadence was self-selected and the load was reduced to the minimum by one of the researchers. All training sessions for both groups were directly supervised by professionals experienced with the training prescription in a ratio of one supervisor per volunteer. During both protocols, standardized verbal stimuli were offered.

### Outcomes Measures

#### Cardiorespiratory Graded Exertion Test

Participants performed a GXT on an electromagnetically braked cycle ergometer (CG04, Inbramed, Brazil) to determine their peak oxygen consumption (VO_2_peak), intensity associated with VO_2_peak (*iV*O_2_peak), and HRpeak. Testing was performed 3–7 days before and after the training period. Briefly, following a 2-min warm-up at 50 W, the load was increased by 25 W every minute until volitional exhaustion, defined as the point at which the participant was not able to sustain a cadence ≥50 rpm. Participants wore a mouthpiece and nose clip, and gas was collected breath by breath by a specific pneumotach connected to the analyzer. VO_2_ and carbon dioxide production (VCO_2_) were analyzed by a metabolic gas collection system (VO2000, MedGraphics, USA) every 10 s. After exhaustion, the load was reduced to 50 W for 2 min of recovery. The highest VO_2_ measured at the cessation of exercise was called VO_2_peak because no participants reached the criteria for VO_2_max (Howley, 2007). To identify *i*VO_2_peak, the highest workload was considered. HR was constantly monitored throughout the test using a HR monitor (Polar RS800, Kempele, Finland). The rating of perceived exertion (RPE) was assessed every minute using the 6–20 Borg scale (Borg, 1982).

#### Anthropometric Measures

Each participant's height and body mass were measured to the nearest 0.1 cm and 0.1 kg, respectively. All anthropometric measurements (3–7 days before and after the training period) were carried out at the same phase of the menstrual cycle (follicular phase) and by the same examiner (to avoid inter-examiner variability), who was previously trained and experienced in these types of measurements and was blinded to group allocation. BMI was calculated from these data. Waist circumference was measured at the level of the smallest circumference above the umbilicus and below the xiphoid appendix (American College of Sports Medicine, 2011).

Five subcutaneous skinfolds (triceps, subscapular, suprailiac, abdominal, and thigh) were measured on the right side of the body using an adipometer (Premier, Cescorf, Brazil) and following the recommendations of the American College of Sports Medicine (2011). The mean of three valid measurements obtained at each skinfold site was used in the analysis. Intraclass correlation coefficient was 0.991 for triceps, 0.993 for subscapular, 0.996 for suprailiac, 0.995 for abdominal, and 0.986 the thigh skinfold. The Typical Error Measurement (TEM) was 0.7 mm for triceps, 0.8 mm for subscapular, 0.7 mm suprailiac, 0.7 mm for abdominal, 1.0 mm for thigh, and 11.2 mm for sum of five skinfolds (Σskinfolds).

#### Dietary Intake Evaluation

Six dietary recalls were applied by a dietitian, with three 24-h food recalls in the first and three in the eighth week. The quantification of the home measures to their equivalent in grams was made according to values of the Table for Evaluation of Food Consumption in Domestic Measures (Pinheiro et al., 2009).

Food intake was calculated in the *Dietpro® Clinical* software, version 5.8.1 (S. SISTEMAS, Minas Gerais, Brazil), using as reference the values of the Food Composition Table (Philippi, 2015), Brazilian Food Composition Table (TACO) (Núcleo de Estudos e Pesquisas em Alimentação, 2011), and United States Department of Agriculture (USDA). Total energy (kcal), carbohydrates (g), proteins (g), and lipids (g) were obtained. After the quantification, the mean initial and final intakes were compared for the results.

### Statistical Analysis

Data were entered into an Excel spreadsheet (Microsoft) and imported into Statistical Package for the Social Sciences (version 20.0; SPSS Inc., Chicago, IL). Based on tests and retests for 49 participants, the standard error of the measurement (SEM) was calculated for triceps, subscapular, suprailiac, abdominal, and thigh skinfolds as previously described (Barbalho et al., 2017). Responsiveness was defined as changes that exceeded two times the SEM in favor of beneficial changes, since this response is supposed to be a true physiological adaptation beyond what might be expected to result from technical and/or biological variability (Barbalho et al., 2017). The responsiveness threshold was set at 0.7 mm for triceps, 0.8 mm for subscapular, 0.7 mm for suprailiac, 0.7 mm for abdominal, 1.0 mm for thigh, and 11.2 mm for sum of skinfolds. Paired *t*-tests were used to compare pre and post values of anthropometry measures, $\overset{{}^{\circ}}{V}$O_2_peak, and *i*$\overset{{}^{\circ}}{V}$O_2_peak within each group. Analysis of covariance (ANCOVA) was used to compare post-intervention values, using baseline values as covariate. Pearson's chi-squared test was used to analyze the distribution of R and NR between groups. Measures of the effect size (ES) for differences were calculated by dividing the mean difference by the standard deviation (*SD*) of the pre-training measurement. The magnitude of the ES was classified according to the following criteria: *d* < 0.2 was considered “trivial,” *0.2*<*d*<*0.5* was considered “small,” *0.5*<*d*<*0.8* represented “medium,” and d > *0.8* constituted “large” (Cohen, 1988). Data are presented as numbers and percentages for categorical variables and are expressed as mean ± *SD*. A significance level of 0.05 was adopted for all statistical tests.

## Results

Adherence to training in HIIT and SIT groups was 76.5 and 74.2%, respectively. Only one participant from each group needed to replace one exercise session at the end of the intervention period. Moreover, one participant from the HIIT group reported vomiting and two participants from the SIT group reported dizziness after a training session.

### Cardiorespiratory Fitness

The HIIT and SIT groups improved (*P* < 0.001) VO_2_peak by 14.5 ± 22.9 and 16.9 ± 23.4%, respectively, as well as *iV*O_2_peak by 6.2 ± 12.2 and 10.6 ± 18.1%, respectively (Table 1). The ANCOVA revealed no significant difference between groups for the changes in VO_2_peak and *i*VO_2_peak (*P* > 0.05).

### Anthropometric Measures

Decreases (*P* < 0.05) in body mass (−1.2 ± 2.6 kg) and BMI (−1.2 ± 2.6 kg.m^−2^) were observed only for the SIT group (Table 1).

**Figure 2:**
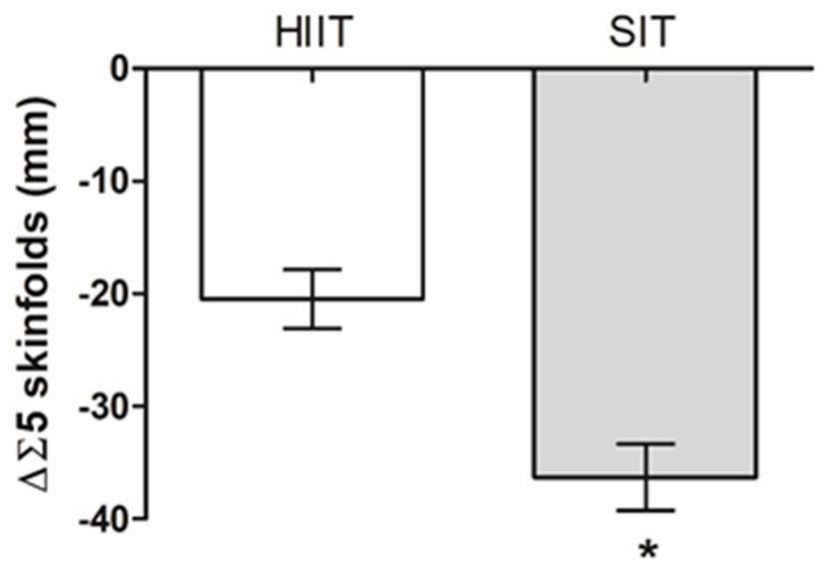
Changes in the sum of skinfolds (ΔΣ5 skinfolds) induced by High intensity interval training (HIIT, *n* = 25) and Sprint interval training (SIT, *n* = 24). Data are expressed as means ± standard deviation*P < 0.05.

The sum of the five skinfolds was reduced by 15.8 ± 7.9 and 22.2 ± 6.4% from baseline (*P* < 0.001) for the HIIT and SIT groups, respectively (Figure 2 and Table 1). The results of ANCOVA revealed that the reductions were greater for SIT than for HIIT. The HIIT and SIT groups significantly decreased (*P* < 0.001) triceps (−13.8 ± 9.4 and −16.4 ± 7.8%), subscapular (−15.7 ± 11.8 and −24.0 ± 10.6%), suprailiac (−13.8 ± 14.9 and −22.0 ± 9.5%), abdominal (−19.8 ± 10.4 and −26.8 ± 6.7%), and thigh skinfolds (−13.6 ± 8.0 and −19.6 ± 8.1%) (Table 1). There were no significant differences between the groups (*P* > 0.05) in triceps, subscapular, suprailiac, and abdominal skinfold reductions. However, decreases in thigh skinfold were greater for the SIT group (*P* = 0.020). Waist circumference was reduced (P < 0.001) for the HIIT (−3.1 ± 1.1%) and the SIT groups (−3.3 ± 1.8%), with no significant difference (*P* = 0.739) between groups (Table 1).

### Dietary Intake

No significant difference (*P* > 0.05) was found in dietary intake between the HIIT and SIT groups at baseline and after 8 weeks of training. In addition, no significant difference was found after the intervention period for both groups (Table 2).

**Table 2:** Dietary intake of participants before and after 8 weeks of exercise training.

	**HIIT (*****n*** = **25)**				**SIT (*****n*** = **24)**				**Between groups**
	**Pre**	**Post**	**ES**	***P***	**Pre**	**Post**	**ES**	***P***	***P***
Energy intake (kcal)	1594.6 ± 429.9	1577.8 ± 424.9	−0.039_(trivial)_	0.894	1442.8 ± 657.1	1420.6 ± 384.8	−0.041_(trivial)_	0.863	0.243
Carbohydrate (g)	184.7 ± 67.5	186.1 ± 65.8	0.021_(trivial)_	0.930	171.9 ± 79.6	163.5 ± 56.6	−0.121_(trivial)_	0.620	0.258
Protein (g)	75.5 ± 23.8	69.9 ± 20.4	−0.252_(small)_	0.272	64.4 ± 21.6	65.6 ± 21.6	0.055_(trivial)_	0.822	0.893
Lipids (g)	61.4 ± 20.2	61.3 ± 22.9	−0.004_(trivial)_	0.988	53.9 ± 33.0	55.9 ± 16.9	0.076_(trivial)_	0.762	0.420
Monounsaturated fat (g)	17.1 ± 6.8	16.6 ± 8.0	−0.067_(trivial)_	0.824	14.7 ± 10.9	15.5 ± 6.6	0.088_(trivial)_	0.729	0.645
Polyunsaturated fat (g)	9.3 ± 5.0	9.5 ± 4.1	0.043_(trivial)_	0.881	9.9 ± 6.3	10.2 ± 4.8	0.053_(trivial)_	0.876	0.594
Saturated fat (g)	17.0 ± 7.2	16.3 ± 6.8	0.099_(trivial)_	0.726	14.6 ± 10.7	14.8 ± 4.3	0.024_(trivial)_	0.946	0.469
Calcium (g)	501.7 ± 266.5	536.8 ± 235.6	0.139_(small)_	0.616	572.2 ± 392.9	488.3 ± 193	−0.271_(small)_	0.242	0.322
Sodium (g)	43.6 ± 159.1	12.1 ± 4.9	−0.279_(small)_	0.691	11.8 ± 7.1	11.7 ± 6.2	−0.015_(trivial)_	0.571	0.362
Dietary fiber (g)	1539.9 ± 730.9	1628.5 ± 566.1	0.135_(trivial)_	0.192	1575.6 ± 962.9	1446.7 ± 781.9	−0.146_(trivial)_	0.996	0.884

*Data are expressed as means ± standard deviation. p (values) for within-group (time) effect and interaction (time × group) effect. ES, effect size; HIIT, high-intensity interval training; SIT, sprint interval training*.

### Responders and Non-responders

**Figure 3:**
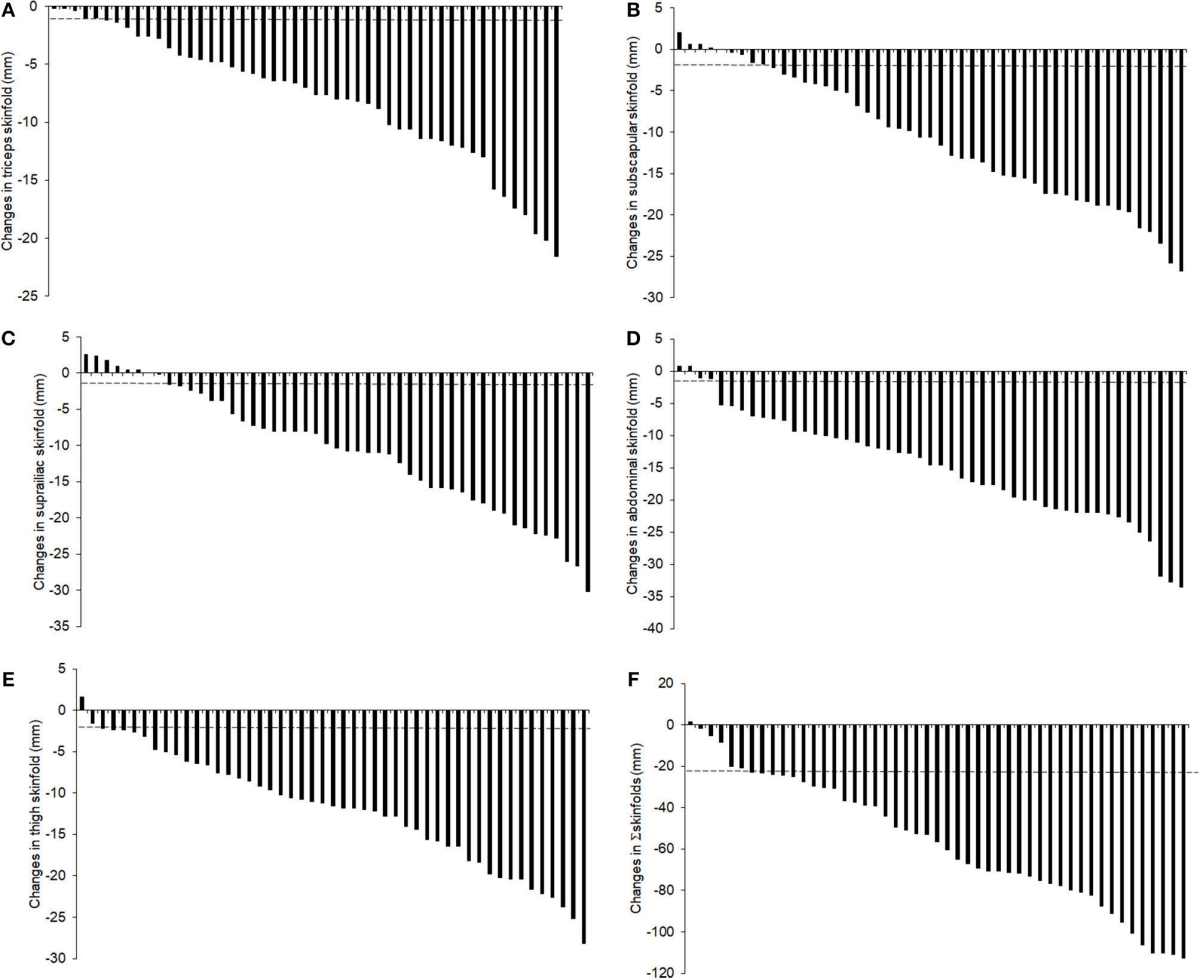
Histogram of the relative changes in triceps **(A)**, subscapular **(B)**, suprailiac **(C)**, abdominal **(D)** thigh **(E)**, and Σ5 skinfolds **(F)** for each individual after 8 weeks interval training in young women.

Forty-one participants were classified as responders (R) to triceps skinfold (20 in the HIIT and 21 in the SIT group), 41 to subscapular skinfold (20 in HIIT and 21 in SIT), 44 to suprailiac (21 in HIIT and 23 in SIT), 45 to abdominal (21 in HIIT and 24 in SIT), 47 to thigh (23 in HIIT and six in 24), and 43 to ∑skinfolds (20 in HIIT and 23 in SIT) (Figure 3). The SIT group presented more R in abdominal skinfolds when compared with the HIIT group; however, no significant difference was found (*P* > 0.05) in the prevalence of R between HIIT and SIT protocols for triceps, subscapular, suprailiac, thigh, and ∑skinfolds.

## Discussion

To the best of our knowledge, this is the first study to compare the effects of HIIT and SIT on anthropometric measures and cardiorespiratory fitness in healthy young females. Our results suggest that 8 weeks of HIIT and SIT improve markers of body fatness and cardiorespiratory fitness, even in the absence of changes in dietary intake. However, our results suggest that the SIT protocol is more efficient than the HIIT protocol for some parameters. In addition, we found greater prevalence of responders for abdominal and suprailiac skinfolds in the SIT group than in the HIIT group.

In agreement with previous studies showing that different forms of IT significantly increased VO_2_max (Gibala et al., 2006; Wisløff et al., 2007; Bacon et al., 2013; Sloth et al., 2013; Gist et al., 2014), the present study found that 8 weeks of HIIT and SIT increased VO_2_peak by 14.5 and 16.9%, respectively. Considering that low cardiorespiratory fitness is a strong independent risk factor for cardiovascular disease and all-cause mortality (Kodama et al., 2009; Barry et al., 2014) and that “lack of time” is a common barrier to regular exercise adoption (Weston et al., 2013), IT might help to increase exercise adherence. While the general recommendations suggest a minimum of 150 min of moderate aerobic activity or vigorous exercise for 75 min per week (World Health Organization, 2010), we found that with only 23 min of SIT performed three times per week, it is possible to increase cardiorespiratory fitness. Moreover, the increases for both groups were similar to those found in previous studies involving protocols with longer durations (Scribbans et al., 2016).

The cardiorespiratory fitness increases observed in the present study are similar to those reported in previous IT interventions (Trapp et al., 2008; Macpherson et al., 2011; Bagley et al., 2016; Higgins et al., 2016). Bagley et al. (2016) submitted 17 women and 24 men to a SIT protocol (4 x 20 s sprints on a cycle ergometer at 175% VO_2_max followed by 2 min of active recovery, three times per week for 12 weeks) and found VO_2_max increased by 18.7 and 6.0% for women and men, respectively. In the study of Higgins et al. (2016), 52 inactive, overweight/obese young women performed one of two experimental interventions: SIT (5–7 x 30 s sprints “*all out*” followed by 4 min of active recovery) and continuous cycling at 60–70% of heart rate reserve. After 6 weeks, the SIT group increased VO_2_peak by 14.1%. The study of Macpherson et al. (2011) involved men and women (*n* = 10 per group) training three times per week for 6 weeks with SIT (30 s all-out running sprints on a manually driven treadmill, four to six bouts per session, 4 min of recovery per bout) vs. MICT (65% VO_2_max for 30 to 60 min). After the intervention, the SIT and MICT groups showed increases of 11.5 and 12.5% in VO_2_peak. These studies indicate that SIT, involving cycling and running, provides an efficient stimulus to improve aerobic metabolism despite its short duration. An important aspect is that the previously mentioned studies (Macpherson et al., 2011; Bagley et al., 2016; Higgins et al., 2016) also used active recovery. Active recovery contributes to increased aerobic metabolic activity and can also influence performance (Buchheit and Laursen, 2013). It appears that active recovery may decrease muscle oxygenation (Buchheit et al., 2009) and impair PCr resynthesis (Spencer et al., 2006). In addition, the active recovery might decrease the performance of the next effort when the intensity is ≥ 45% iVO2max). Therefore, if active recovery is chosen, it should last at least 3–4 min at a low intensity (Belcastro and Bonen, 1975) to allow maintenance of the high intensity of exercise during the following interval.

Both types of IT promoted reductions in body mass, markers of subcutaneous fat (skinfolds) and waist circumference, which is in agreement with the suggestion of Astorino and Schubert (2018) that HIIT and SIT increase whole-body fat oxidation. An important aspect of the present study is that the participants did not present a statistically significant difference between the groups in the pre-intervention period. In addition, our results were similar to those of Hazell et al. (2014), who reported that 6 weeks of a running SIT protocol of similar intensity and duration to the one used in our intervention reduced fat mass by 1.2 kg with 0.5 kg reduction in body mass despite no changes in dietary intake.

Previous studies using different forms of IT with longer periods of training also found fat loss in postmenopausal women with type II diabetes (Maillard et al., 2016), inactive young women (Trapp et al., 2008; Panissa et al., 2016), overweight men and women (Heydari et al., 2012; Higgins et al., 2016), and mixed samples of young men and women (Tremblay and Bouchard, 1994; Macpherson et al., 2011). One of the few studies comparing different IT protocols (Tong et al., 2018) compared the effects of SIT and HIIT in reducing abdominal visceral fat in 46 obese women. The participants were assigned to one of three experimental groups: SIT (6 s all-out sprint followed by a 9 s passive recovery for 80 cycles), HIIT (4 min exercise bouts at an intensity of 90% VO_2_max, followed by a 3-min passive recovery). and Control group (no exercise). After 12 weeks, there was a reduction in abdominal visceral and subcutaneous fat. However, SIT group had lower reduction in subcutaneous abdominal fat (−17.4 vs. 40.7 cm^2^) and trunk fat mass (1.2 vs. 2.0 kg) than HIIT group. No difference was found between SIT and HIIT for visceral abdominal fat, total fat mass, gynoid, and android fat mass. Probably, the difference between the results present by Tong et al. (2018) and our study was related to training protocol, since the short duration of SIT in the study by Tong et al. (6 vs. 30 s) might have lead to a less pronounced effect on fat metabolism and post exercise energy expenditure (Islam et al., 2017).

It is important to note that, according to our results, women reduced the Σskinfolds without changes in dietary intake, which is similar to results previously reported by Zhang et al. (2017) and Trapp et al. (2008), who observed changes in body composition after intervention with IT without changes in dietary intake. Several studies have suggested that the increases in post-exercise fat oxidation seem to be influenced by glycogen depletion (Withers et al., 1991; Kiens and Richter, 1998), and protocols that rely more on the glycolytic system might be more advantageous in this aspect (Whyte et al., 2013; Tucker et al., 2016). The higher reduction in the sum of skinfolds promoted by SIT might be due an increased oxidation of fat during the rest period, as previously reported (Withers et al., 1991). In agreement with this, previous studies have shown that IT protocols that lead to glycogen depletion result in increased fat oxidation (Withers et al., 1991; Kiens and Richter, 1998; Whyte et al., 2013; Tucker et al., 2016). Therefore, it appears that restoration of glycogen has a metabolic priority during recovery, leading to an increase in fat oxidation (Kiens and Richter, 1998).

One important aspect of the present study is that training was performed in a standard fitness facility using commercially available stationary bicycles, which is important to its practical application. However, one important aspect that limits the generalization of our results is that our training sessions were closely supervised at a 1:1 ratio. Considering that previous studies show that the results of an exercise intervention depend on supervision (Gentil and Bottaro, 2010; Knab et al., 2011; Ramírez-Campillo et al., 2017), the current findings might not be reproducible in unsupervised situations. Another apparent limitation is that our study did not identify the responders and non-responders to VO_2_peak. In addition, lacks a control group that did not perform any type of exercise and the lack of a more accurate instrument for measuring body composition. However, since the participants did not change their nutritional habits and were evaluated at the same phase of the menstrual cycle, seasonal variations are unlikely to have been able to alter the results. As for the skinfolds measures, whilst we recognize that it might be a limited method to estimated body composition, it has been shown to be a highly reproduceable and widely used method (Jackson et al., 2009; Silva et al., 2009; Alves et al., 2017; Astorino et al., 2018); therefore, it is our opinion that it might be suitable to access the changes induced by an intervention on markers of body fatness.

## Conclusion

Both HIIT and SIT protocols increased cardiorespiratory fitness and promoted reductions in adiposity indicators in healthy young women, even in the absence of dietary changes. Moreover, the SIT protocol induced greater improvements in some markers of body fatness than the HIIT protocol.

Considering the low physical activity levels in the population, the high prevalence of excessive body fat, and the fact that lack of time is a common barrier to exercise adoption (Weston et al., 2013; Vella et al., 2017), both protocols appear to be viable alternatives, since HIIT and SIT protocols lasted 33 and 23 min, respectively. In addition, one advantage of SIT is that it does not need complex tests to define the intensity of the exercise, which might contribute to its widespread use in cases where no clinical contraindications exist.

## Author Contributions

JN and PG conceived and designed the research. JN, PL, and JdO performed experiments. JN, RV, and PG analyzed data. JN, RV, AR, CdL, GP, RR-C, and PG interpreted results of experiments. JN and PG drafted manuscript. AR, CdL, GP, RR-C, and PG edited and revised manuscript. All authors approved final version of manuscript.

### Conflict of Interest Statement

The authors declare that the research was conducted in the absence of any commercial or financial relationships that could be construed as a potential conflict of interest. The handling Editor declared a past co-authorship with the authors RR-C and PG.
